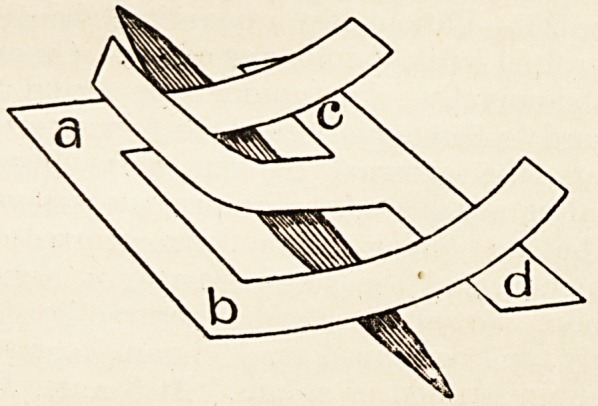# The Treatment of Everyday Cases

**Published:** 1910-04-02

**Authors:** G. R. Bickerstaff


### The General Practitioner's Column.

[Contributions to this Column are invited, and, if accepted, will be paid for.]

### THE TREATMENT OF EVERYDAY CASES.

By G. R. BICKERSTAFF, M.R.C.S., L.R.C.P.

A country doctor's surgery does not often appear, to anyone fresh from hospital, a suitable place for minor operations and dressings, yet some very fair results are generally obtained there, and with scanty materials. It teaches one to make the best of what is at hand, and to learn to work with few and simple materials in a very limited space.

Any morning we may find some wounds requiring immediate treatment in the short time before starting on the morning round.

For example, a *clean incised wound*, such as is caused by a bread-knife slipping. We should not encourage the patient to unwrap his hand till we have all things we may want in readiness, as such wounds are apt to bleed freely. So we lay out absorbent wool, some clean bowls or trays, gauze, bandages, artery-forceps, scissors, and a needle or two, threaded with wire, horsehair, or silk, before we unwrap the wound.

If the hand is fairly clean, it is better to keep it dry, and only wipe the surrounding parts and edges of the cut with bits of clean wool. Then, if no vessel large enough to spurt is divided, the cut edges may be brought together with strapping, if they are dry enough for the plaster to stick; or if we cannot make them so, a stitch or two will be required. A pad of alembroth or other gauze firmly bandaged in place is generally sufficient dressing.

The hand, of course, should be kept up in a handkerchief sling if the wound is of any size, and even the slightest cut heals quicker if the limb is supported and at rest.

*Contused or lacerated wounds* caused by machinery often need much cleaning-up of the surrounding parts with carbolic lotion, or preferably, ethereal soap solution, or germicidal soap and water.

Stitches may often be avoided by using carefully-cut and well-warmed tongue-pieces of strapping to bring the edges of the wound together, as shown in the diagram. The long edges *a* to *b*, and *c* to *d*, must be pressed down first on the dried edges of the wound, and made to adhere firmly before drawing on the tongues to bring the wound-edges together.

Friar's Balsam (Tinct. Benzoin. Co.) is a most useful application for small wounds of all kinds. The only drawback is the smarting, which soon passes off, however. A pad of lint moistened with this drug forms, upon drying, a waterproof, germ-proof covering, which adheres firmly, and therefore is very useful for children's cuts and for scalp-wounds. When the pad has to be removed in a few days for re-dressing, patiently soaking and snipping little bits with scissors will soon bring it off without damage to the healing wound beneath. Medicated gauze (sal alembroth, cyanide, etc.), forms a very good pad, too, but this also needs soaking before removal.

A greasy application such as carbolic oil, or weak antiseptic ointment gives no trouble in removal, but it is also more liable to slip out of place, and, I think, never gives such good results in healing. I always carry a small phial of friar's balsam in a pocket dressing-case; and have found it most useful as a general emergency-dressing.

### The Common Cold.

Patients at this time of year are frequently complaining of "colds." A "cold in the head" is a most common ailment, and one which is well worth a little care in avoiding, or in cutting short if already too far established.

In most people the first symptoms are slight feverishness, and commencing sore throat. Perhaps the throat is not exactly sore, but feels "stiff" on one side, and dry. Now, if a little Dover's powder and acetosalicylic acid are taken—probably one or two doses will be sufficient—further developments may be checked. Five grains of each at bedtime will usually be enough for a dose; large doses, especially if repeated, may cause headache and constipation. Aperients will probably be required also.

When the cold has not been "taken in time," and is established in the eyes and nose with more or less profuse watery secretion, a good deal of relief can be still obtained from the above drugs, combined with menthol to smell, either in the form of compound menthol snuff, or carried in a small smelling-bottle or phial, frequently used, and carefully corked again, as it soon evaporates. Common peppermint lozenges, "extra strong," sucked frequently, are very comforting to some sufferers, and, when they can be indulged in without annoyance to others, have proved a good remedy on account of the menthol they contain. Menthol lozenges, of course, may be purer, but are generally less pleasant to taste.

We must try to keep in a uniform temperature as much as possible, not in a hot room, for this increases the discomfort, but avoiding especially wind and draughts. A good hot drink—hot milk, gruel, or water as hot as it can be sipped—at bedtime each night, and plenty of bed-clothing will help towards cure. Artificial light and a hot fire always bring out the discomforts of a cold, so it is better to retire early.

When the cold is passing down to the chest, paregoric (Tinct. Camph. Co.) is most useful—20 or 25 minims for a full dose, in water. It may be advantageously combined with five minims of ipecacuanha wine, or the same dose of antimonial wine.

The latter, according, to De Styrap, has a more soothing effect on the bronchial mucous membrane, besides helping to lower the feverishness. A pleasant form of taking the two drugs would be in the form of a linctus, such as:—

| | |
|---|---|
| Tinct. Camph. Co. ... ... ... ... | ♏xx. |
| Vin. Antimonal ... ... ... ... | ♏v. |
| Syrup Limonis ad ... ... ... | ʒj. |

A teaspoonful every three hours.

## Figures and Tables

**Figure f1:**